# Web-based public health geographic information systems for resources-constrained environment using scalable vector graphics technology: a proof of concept applied to the expanded program on immunization data

**DOI:** 10.1186/1476-072X-5-24

**Published:** 2006-06-03

**Authors:** Raoul Kamadjeu, Herman Tolentino

**Affiliations:** 1 Pubic Health Informatics Fellow, Division of Public Health and Informatics, Epidemiology Program Office, Centers for Disease Control and Prevention (CDC), Atlanta, GA, USA; 2 National Immunization Program, Global Immunization Division, Global Measles Branch, Centers for Disease Control and Prevention (CDC), Atlanta, USA; 3 National Immunization Program, Epidemiology and Surveillance Division, Vaccine Safety Branch, Centers for Disease Control and Prevention (CDC), Atlanta, GA, USA

## Abstract

**Background:**

Geographic Information Systems (GIS) are powerful communication tools for public health. However, using GIS requires considerable skill and, for this reason, is sometimes limited to experts. Web-based GIS has emerged as a solution to allow a wider audience to have access to geospatial information. Unfortunately the cost of implementing proprietary solutions may be a limiting factor in the adoption of a public health GIS in a resource-constrained environment. Scalable Vector Graphics (SVG) is used to define vector-based graphics for the internet using XML (eXtensible Markup Language); it is an open, platform-independent standard maintained by the World Wide Web Consortium (W3C) since 2003. In this paper, we summarize our methodology and demonstrate the potential of this free and open standard to contribute to the dissemination of Expanded Program on Immunization (EPI) information by providing interactive maps to a wider audience through the Internet.

**Results:**

We used SVG to develop a database driven web-based GIS applied to EPI data from three countries of WHO AFRO (World Health Organization – African Region). The system generates interactive district-level country immunization coverage maps and graphs. The approach we describe can be expanded to cover other public health GIS demanding activities, including the design of disease atlases in a resources-constrained environment.

**Conclusion:**

Our system contributes to accumulating evidence demonstrating the potential of SVG technology to develop web-based public health GIS in resources-constrained settings.

## Background

The geography of disease has been dramatically improved by information technology since the map of cholera cases in Soho by John Snow [1]. The proliferation of computers, associated with the rapid growth in the number of Geographic Information Systems (GIS) tools, has made spatial analysis of pathological factors and their relationship to the environment a common practice in public health research [2,3]. GIS are useful for visually analyzing epidemiological data and revealing trends and relationships that may be hidden in a tabular form view. The many GIS tools range from simple free mapping software to highly sophisticated applications, usually based on proprietary technology and data formats. However, GIS technology requires considerable skills in order to be fully operational and, for this reason, is sometimes understood and used by only a few specialists within the public health community. Meanwhile, the need to access geospatial information has grown among public health professionals, policy makers, managers, researchers, students and the general public [3,4]. Recently, web-based GIS have emerged as a solution to allow the access of geospatial information to a wider audience with limited computer and GIS knowledge [3,5,6]. The publication and distribution of spatial data are increasingly important activities enabling organizations to share maps as images over the Internet. We used Scalable Vector Graphics (SVG) [3,7] to develop a database-driven web-based GIS. We applied this system to Expanded Program on Immunization (EPI) data from three countries of WHO AFRO (World Health Organization – African Region). In this paper, we summarize our methodology and demonstrate the potential of a free and open standard to contribute to the dissemination of EPI information by providing interactive maps to a wider audience through the Internet.

EPI was launched by WHO in 1977 with the objective of immunizing 80% of the world's children against six of the most deadly vaccine-preventable diseases [8]. The program involves a coalition of partners (governments, international organizations, non-governmental organizations, and religious agencies) and promotes the use of data for action. GIS is now an integral component of EPI management and is used to evaluate and plan immunization activities. More specifically, GIS in EPI is used to visually display and compare immunization coverage data among districts, regions, and countries (thematic maps) and to track changes in disease location (dot density maps). The operational unit of EPI activities is the health district, the boundaries of which may or may not coincide with administrative subdivision boundaries. To monitor country immunization coverage, choropleth mapping is the preferred approach. In EPI choropleth (thematic) maps, coverage data that fall within a specific class interval are assigned a unique color code. The standard color codes used in WHO AFRO are: red for immunization coverage below 50%, yellow for coverage between 50% to 80% and green for coverage above 80%. Mapping in this context is viewed mostly as a tool to communicate information effectively to the immunization stakeholders.

The two main data structures for representing graphics information on computer are raster and vector graphics [9]. In raster graphics, an image is represented as a rectangle of picture elements or pixels. Each pixel is represented by its Red Green Blue (RGB) color values. The series of pixels is termed a bitmap and can be stored in a compressed format (JPEG, GIF, and PNG). In a vector graphic system, the image is described as a series of geometric shapes. A vector-viewing program draws shapes at specified sets of coordinates.

The classification of web-based maps is widely discussed elsewhere [10]. This classification is generally based on how the map is produced and responds to users' interactions. A distinction is made between static and dynamic maps with further subdivisions into view-only maps and interactive maps. The most common type of map found on the Internet is the static view-only map. This is sometimes a scanned cartographic image stored as a bitmap. A dynamic map on the contrary allows change in one or more of its spatial data component, the incorporation of links, and other fine tuning functions (mouse roll-over, mouse click) controlled by the user. Several technologies can be used to create and animate dynamic maps including the proprietary Flash Macromedia^® ^technology [11], Vector Markup language (VML) [12] and Scalable Vector Graphics (SVG).

SVG is used to define vector-based graphics for the internet using XML (eXtensible Markup Language). SVG is an open, platform-independent standard maintained by the World Wide Web Consortium (W3C) [13]. SVG became a W3C recommendation in January 2003 under SVG 1.1 and was designed to integrate with other W3C web standard efforts like Xlink, XML Namespaces, Document Object Model (DOM), Cascading Style Sheet (CSS) and Extensible Style sheet Language (XSL). A SVG document contains three main components: the XML declaration tells the parser that the incoming document is a XML document; the Document Type Declaration (DTD) identifies the type of XML document which in this case is an SVG document. The SVG document itself is contained between an opening and closing <svg> tag. SVG contains some predefined shape elements (rectangle, circle, line polygon, path etc.) that can be used alone or in combination to produce simple to highly sophisticated graphics [9,14,15]. Since SVG is XML, it will not be recognized by some browsers and should therefore be embedded into web pages.

Another important component of SVG technology is the SVG viewer. The viewer interprets and renders the SVG document. The viewer can be installed in a web browser as a plug-in such as Adobe SVG Viewer^® ^[3] for Internet Explorer^®^, or can be provided as a built-in component (Mozilla Firefox). A comprehensive list of SVG viewers is maintained by W3C [16].

## Results

**Figure 1:**
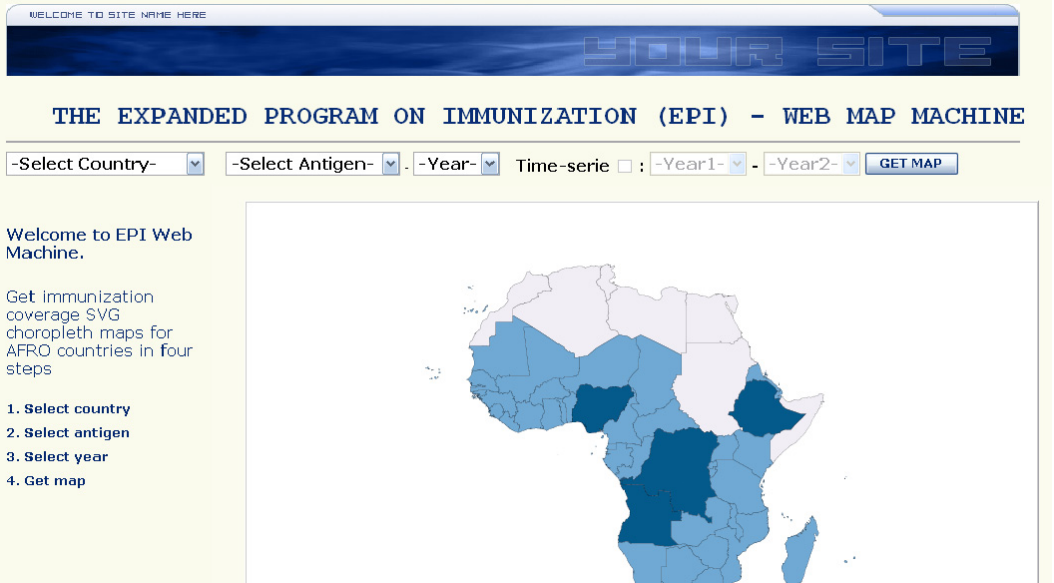
**System main screen**. The main system allowed the user to select the country, the antigen and the period to map. The complete demo was optimized for Internet Explorer version 5 or higher using Adobe SVG Viewer version 3.03 on a 1280 × 1024 screen display.

**Figure 2a:**
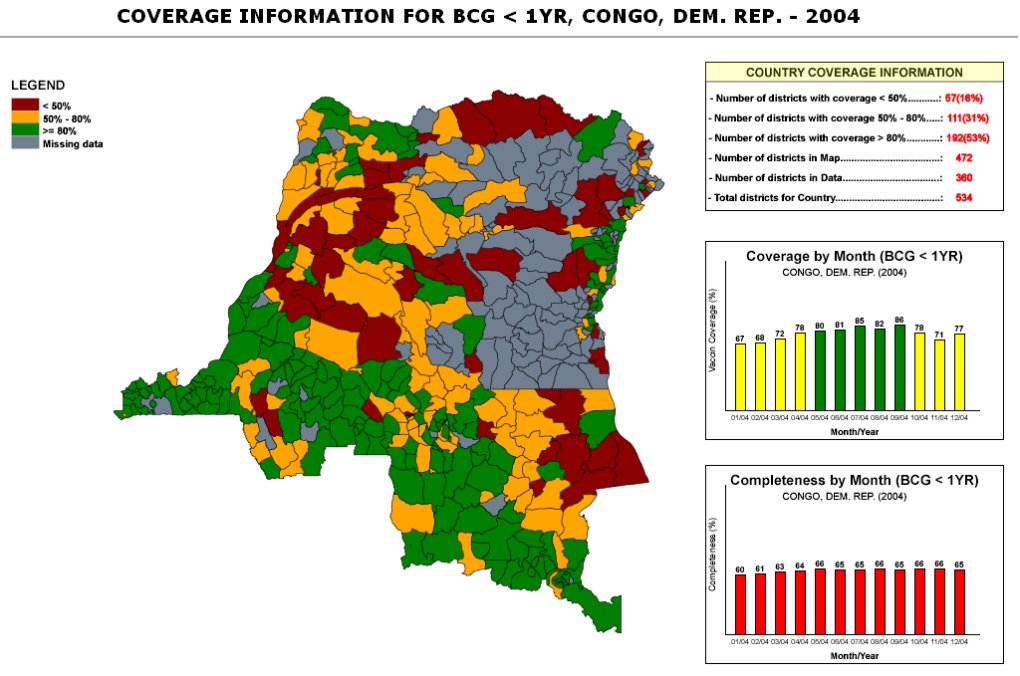
**System output (map)**. The output combines a thematic map of vaccine coverage with country monthly coverage graph.

**Figure 3:**
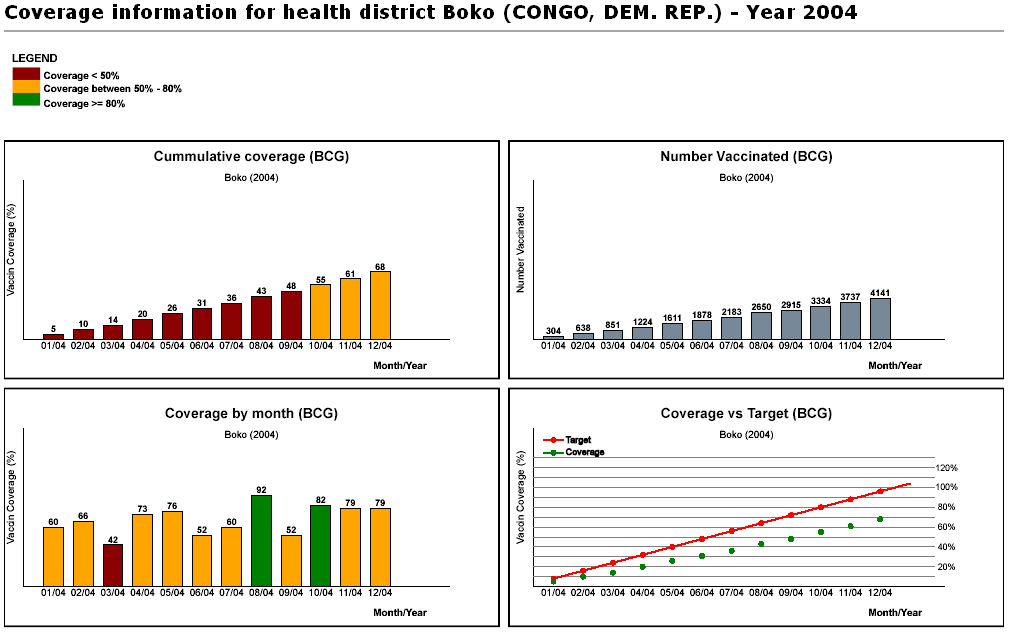
**System output (graph)**. Additional information such as district immunization performance (e.g.: target versus actual graph by month) will popup on users interactions (mouse click, mouse roll-over) with district bar graph.

Based on information entered by the user via a simple interface (country, antigen, and year or time interval) (Figure 1), the system generates interactive district level country coverage maps and graphs. The web-based GIS described in this paper is an EPI map machine for use by non-GIS skilled end-users [17]. The system displays SVG immunization coverage maps at the district level for the selected country, antigen, and year. The output of the system (figure 2a and 3) consists of a thematic map showing country immunization coverage at the district level for the specified antigen and period. The legend displays three categories which are consistent with WHO standard color codes to represent vaccine coverage. An additional color category (grey) was added to represent districts without available coverage data in the database. User interactions with the map include the ability to display the name and coverage value for a district on mouse rollover, and the ability to link to more specific district information when clicking on a specific bar graph. At this point, additional functions can be implemented including export to different formats such as Geographic Markup Language (GML) [18] and Portable Network Graphics (PNG) file, Adobe PDF file or to the printer. The system does not require specific training or knowledge of GIS technology. Combining different but complementary summarization tools like maps and graphs on the same display area greatly enhance the quality of the information displayed as demonstrated by several SVG public health projects [19-22].

## Discussion

The benefits of SVG technology in computer graphics and in mapping have been demonstrated and are summarized in table 1. Although this technology is quite new, we foresee that the number of GIS web-based systems using this technology will grow as the technology matures and the need to exchange GIS data with a wider audience increases.

**Table 1:** Advantages of using SVG in graphics design

• Pure text (can be modified by a wide range of simple tools) and is searchable
• Smaller and more compressible than image files
• Scalable (do not lose quality when zoom or resize maps)
• High quality printing at any resolution
• Open standard (not proprietary so is freely available)
• Pure XML, human readable
• Animation possible by combining with *JAVASCRIPT*
• Support from major industries (e.g. IBM, Microsoft, Adobe)
• A W3C recommendation. To work with other W3C recommendations such as CSS (Cascading Style Sheets), DOM (Document Object Model) and SMIL (Synchronized Multimedia Integration Language)

Some of the factors that could contribute to the generalization of web-based GIS for EPI include a long tradition of data sharing, the availability of routine immunization data of good quality and the growing interest of donors and the general public in global immunization activities. The approach describe here can be applied to other public health activities such as disease surveillance. This work also contributes to existing efforts to improve web-based public health GIS and disease atlases.

The objective of this work was to demonstrate the use of SVG technology to design a web-based GIS for EPI and does not address the relative value of using SVG over other approaches. Some factors that may influence the choice of a web-based GIS development platform include cost and level of expertise available. Not ignoring the relationship that can exist between these two parameters, the use of an open standard like SVG is mostly intended to address the former.

As with all new Internet technologies, a balance should be made between the strengths brought and the weaknesses associated with early implementation. SVG is not completely supported by web browsers and may not fully interact with existing technologies. Easy access to SVG source code may be a matter of concern for developers; meanwhile draft documents for SVG 2.0 indicates that encryption may be integrated in SVG 2.0 [23]. Existing SVG viewers provide varying levels of implementation of W3C recommendations; this sometimes results in inconsistent display of SVG graphics across browsers. The system described in this paper was optimized for Internet Explorer Adobe SVG Viewer version 3.03 and was not fully tested with other existing viewers. However, these limitations will probably be overcome in the near future as intensive work is being conducted by W3C, SVG community and major industries [22].

## Conclusion

This paper provides an example of integrating SVG technology in the design of a web-based GIS applied to EPI data. The resulting graphical display and enhanced system responsiveness position SVG as a valuable technology in the design of public health GIS and atlases in resources-constrained environment. Moreover, integrating SVG technology in web-based GIS will bring public health maps to a wider community of users with limited GIS and computer knowledge.

## Methods

### System overview

**Figure 4:**
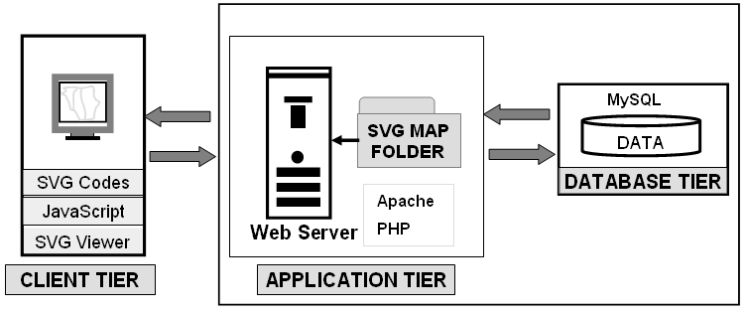
**System architecture**. The system used the *PHP 5.04*, Apache/1.3.33 (Win32) and MySQL 4.1.10a-nt.

The system was designed using a 3-tier architecture (figure 4) consisting of a client tier (web browser), an application tier built on Apache [24] and *PHP *[25], and a database tier running on MySQL [26].

The client tier gets input from the user and displays the final output. It consists of the SVG maps, graphs and table with embedded *JAVASCRIPT *and Xlink which implement interactive functions. Our system was optimized for Internet Explorer^® ^with Adobe SVG Viewer^® ^plug-in. Taking advantage of the specific characteristics of vector graphics we have implemented several operations at this level such as link operations which can be performed on both the map and the graphs.

The application tier is made up of an Apache web server with the web scripting language *PHP*. It interfaces between the database tier and the client tier. The processing at this level involves selecting the appropriate SVG map from the map repository, passing user's queries to the immunization database, getting the results from the immunization database, formatting the SVG map and graphs based on the results of the query and sending the final output back to the client tier for display.

**Figure 5:**
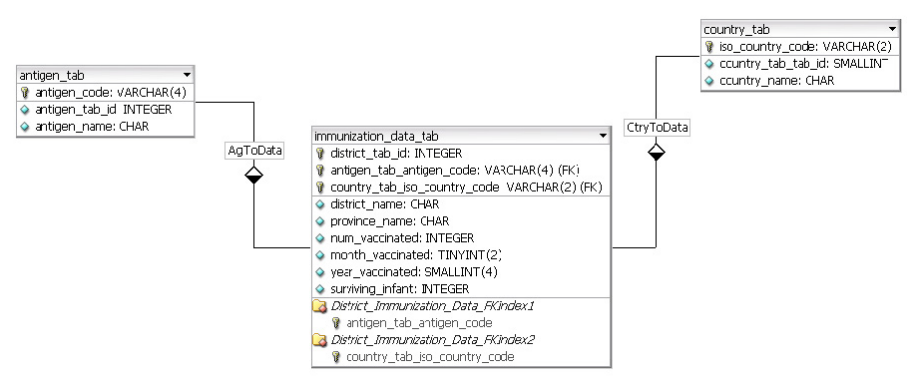
**Database structure**. The database was designed to minimize the need to reformat the original dataset.

The database tier contains immunization information. For each district, information required to calculate vaccination coverage included the total number of children vaccinated with a specific antigen and the number of surviving infants (target population) for the district. Figure 5 displays the database structure and the array elements resulting from the process of a user query. Incoming queries from the application tier calculate immunization coverage by district and return the corresponding color code back the application tier where it is further integrated in the fill property of the district path.

### System design

The first step in designing the system was to create a repository of SVG country maps at the district level. Although SVG documents can be created and edited with basic text editors, a translator is necessary to convert the massive amount of information required to build a map. District level country maps ESRI shape files were loaded into MapInfo Professional and converted to SVG using Map2VSG [27] tool. The resulting SVG files were processed to integrated *PHP *functions and additional *JAVASCRIPT *codes. The value of the fill-opacity CSS attribute, corresponding to a color code value (green, red, yellow and grey) was returned by a *PHP *function. Final SVG files were stored as *PHP *files in the map repository. A relational database was created to store district immunization information (e.g. number of children immunized for each district, by month, population data). The database was designed keep the reformatting of the original WHO-AFRO EPI datasets to a strict minimum.

## Competing interests

The author(s) declare that they have no competing interests.

## Authors' contributions

All authors designed the overall architecture of the system. RK prepared the SVG maps and designed the database. HT programmed the main PHP modules. All authors read and approved the final manuscript.

**Figure 6:**
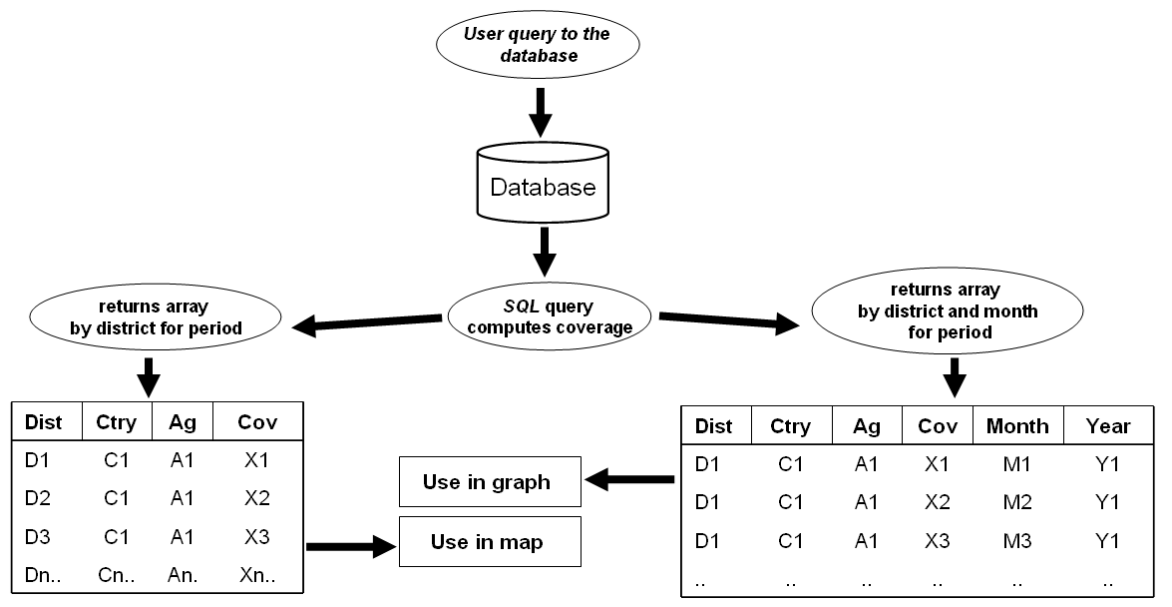
**Data processing**. Vaccination coverage is calculated by the query. Resulting arrays are used to format SVG maps or graphs.
